# Editorial

**Published:** 1860-05

**Authors:** 


### Editorial.

THAT "DECLARATION OF INDEPENDENCE."

Our friend, the publisher of the "American Dental Review," seems not to relish our remarks on his Declaration of Independence. To revenge himself, he brags on our special friend Jno. T. Toland, which infliction we hope to bear with becoming meekness. But we are at a loss to know why he calls Toland our "*sub.*" Is our friend L. "sub" to all those independent editors of his? Verily, if *sub* means *under*, John is not sub to anything, except that he is *under* a fair way to eclipse every body else in the sale of dental materials.

Dr. L. tells us that it is unfortunate we did not *see* somebody beside ourself, and suggests that we might have seen the writer that stigmatized certain parties as "Sutlers of the regular army." Well, we did take *two* straight looks at that fellow, as the pages of the "Register" will manifest.

In conclusion, the Doctor reminds us that he has a great variety of *ripened* experiences, which may be all true enough; but as he is still young, some of these must have matured early. We hope, however, that they are sound at the core—not rotten, or wormeaten, as is often the case with fruits which ripen so much faster than other specimens on the same tree. But as Dr. L. disclaims any wish to intimate that his editors are likely to be more independent than others, we are sorry we said anything. We'll "rub it all out," Doctor. W.

### SENSATIONS ATTENDING ARTIFICIAL DENTURES.

We have occasionally observed conditions attending artificial dentures, particularly in full sets, for which we are unable satisfactorily to account. We will illustrate this subject by giving the experience of a lady who is wearing a full set of teeth.

Mrs. D——, about thirty-five years of age, has been wearing a full set of teeth between three and four years; she experiences some sensations similar to those of the natural teeth, for instance, after eating fruit, or using acids, she experiences that very unpleasant sensation commonly known as the teeth on edge; it is attended with all the unpleasant sensations of that condition upon the natural teeth, amounting sometimes to apparent soreness. Her teeth also sometimes seem as though they were almost ready to ache. Food or foreign substance between them, causing pressure, produces the same unpleasant sensation experienced when a foreign body is between the natural teeth. In this case the plates, which are of gold, fit well in the mouth, and the teeth antagonize very accurately. Now, we might be disposed to charge this somewhat to the imagination, were it not for the fact that this is just the experience of a number of others, which has been given us.

We will be quite thankful to any who will answer two or three questions in regard to such cases, clearly and definitely.

First, What is that condition of the natural teeth, commonly known as the teeth on edge? Second, How is it produced on artificial teeth? Third, How is the sensation of pressure exhibited on artificial teeth on plates? T.

### UNUSUAL PROTRUSION OF THE INFERIOR MAXILLA.

In ——, 1859, I inserted a full set of teeth for Mrs. B—. The relative position of the inferior jaw with the upper was very good. Platina plates were made, which fit the gums well, the teeth were ground on to antagonize very perfectly, according to the ordinary arrangement, the superior anterior teeth closing over the inferior just sufficient for a good antagonism. Molars and bicuspids of both jaws had prominent cusps, so that they locked well together. The patient used the teeth well for masticating in a very short time. Within eight months, however, there was so much elongation or protrusion of the inferior jaw, that the inferior teeth came outside of the superior front teeth, and could not be brought inside by any movement of the jaws the patient was able to make. The whole antagonism was destroyed, indeed, at the end of nine months could not be brought together, and the molar teeth closed at irregular intervals. This produced a fracture of the inferior piece at the median line. In consequence of this derangement, the teeth were deficient in utility. The plates were not changed from their positions upon the gums, nor the teeth upon the plate, except those upon the lower plate, by the fracture referred to, and this could make no material change, for the body held the teeth firmly in their position upon the plate. The teeth of the lower plate were all removed and set in, so as to make proper antagonism with the upper, and it now operates well.

Whether there will be a further protrusion of the jaw, I shall await with patience to learn. Do such cases occur often ? What is the cause ? T.

### THE AMERICAN DENTAL REVIEW.

This journal has made its appearance under its new editorial control. The first number makes a fine appearance,—that is, it looks very much like its predecessors. The three "*nom de plume*" editors appear to work well together. Their "Salutatory" is pretty, apologetic, and short, thus evincing correct taste, modesty and good sense. They hope their "incognito introduction will not be unwelcome to the fraternity ;" and we assure them it will not in this direction. Their signatures are, respectively, D. D. S., M., and A. B. The first may mean that its impersonation is a *Doctor* of *dental surgery*, or that he intends to *do decidedly strong* writing. The second, "M.," is the *middle* editor—the *hub* around which the others revolve. The third is a *bachelor of arts*, and is likely to be *after* the *boys*, so we had better look out.

We like the "Review," and always did ; and we expect to like t still better under the new arrangement. W.

### CONCAVE CORUNDUM WHEELS.

We have been using a new form of corundum wheel, invented by Dr. John G. Hamill, of Lancaster, Ohio. They are made cup-shaped, or rather, they are hemispherical, and about the thickness of the ordinary corundum wheel. When adapted to the shaft, they present on the upper side a large convex surface, and a circular edge or rim of a different form from that of the ordinary wheel; there is also on the inside a concave surface, that can be used if required. This form of wheel adds much to the facility of grinding teeth; it will not supersede other forms, but it is a valuable addition, and one for which the profession is certainly under much obligation to Dr. H. T.

### WEBSTER'S UNABRIDGED ILLUSTRATED DICTIONARY.

We have received a copy of this magnificent work, and have examined it with some care, particularly with reference to the additions made to, and the improvements in this work, and can not but express our gratification that so noble a work has been accomplished. It has all along been received as the standard lexicon, wherever the English language is spoken. To say that the work is perfect, is more than we can do of any human work, yet it meets the requirements as fully as we can well conceive. It is a work that none can be without, and be just to himself. The illustrations are well executed, and many of them are very striking; they make an impression upon the mind, especially of young persons, that is far more permanent than can be made by any ordinary definitions or even description. There is also added a large range of synonyms, which add much to the value of the work. There are other additions, too, for which it should be highly prized. The work is neatly and substantially gotten up, as much so at least as any work of that kind can be. It has been suggested that it should have been published in two volumes, but the student would find that arrangement very inconvenient, having to turn so constantly from one to the other. T.

